# Influence of Ambient Temperature on Part Distortion: A Simulation Study on Amorphous and Semi-Crystalline Polymer

**DOI:** 10.3390/polym14050879

**Published:** 2022-02-23

**Authors:** Anto Antony Samy, Atefeh Golbang, Eileen Harkin-Jones, Edward Archer, Monali Dahale, Alistair McIlhagger

**Affiliations:** Engineering Research Institute, Ulster University, Shore Road, Newtownabbey BT37 0QB, Co. Antrim, UK; e.harkin-jones@ulster.ac.uk (E.H.-J.); e.archer@ulster.ac.uk (E.A.); m.dahale@ulster.ac.uk (M.D.); a.mcilhagger@ulster.ac.uk (A.M.)

**Keywords:** fused deposition modeling (FDM), polymers, warpage, residual stress, finite element analysis (FEA)

## Abstract

Semi-crystalline polymers develop higher amounts of residual stress and part distortion (warpage) compared to amorphous polymers due to their crystalline nature. Additionally, the FDM processing parameters such as ambient temperature play an important role in the resulting residual stresses and part distortion of the printed part. Hence, in this study, the effect of ambient temperature on the in-built residual stresses and warpage of amorphous acrylonitrile-butadiene-styrene (ABS) and semi-crystalline polypropylene (PP) polymers was investigated. From the results, it was observed that increasing the ambient temperature from 50 °C to 75 °C and further to 120 °C resulted in 0.22-KPa and 0.37-KPa decreases in residual stress of ABS, but no significant change in the amount of warpage. For PP, increasing ambient temperature from 50 °C to 75 °C led to a more considerable decrease in residual stress (0.5 MPa) and about 3% increase in warpage. Further increasing to 120 °C resulted in a noticeable 2 MPa decrease in residual stress and a 3.4% increase in warpage. Reduction in residual stress in both ABS and PP as a result of increasing ambient temperature was due to the reduced thermal gradients. The enhanced warpage in PP with increase in ambient temperature, despite the reduction in residual stress, was ascribed to crystallization and shrinkage.

## 1. Introduction

Fused deposition modeling (FDM) is one of the additive manufacturing (AM) techniques that has the ability to 3D print complicated shaped geometries by extruding molten material layer by layer on a pre-heated bed [1,2,3]. In FDM, the feedstock is supplied into the heated nozzle, which then extrudes the filament [4,5]. The deposition process is performed accordingly with a programmed tool path (g-code) of the designed 3D CAD model [6,7]. Once deposited, the filaments are cooled until they reach the ambient temperature. Since the parts in FDM are fabricated layer by layer, the initially deposited layer undergoes cooling, while also being reheated by the subsequent deposited layer. The following layers experience a similar effect due to the continuous deposition process. Furthermore, the parts cool from outward to inward, leading to a non-homogenous cooling. Due to this large thermal gradient difference between the part and the ambient temperature, internal thermal residual stresses are induced, resulting in thermal contraction, otherwise commonly known as shrinkage [8,9]. The part distortion of a 3D printed part can be minimized by optimizing various processing parameters such as print bed temperature [10,11], ambient temperature [12,13], layer thickness [14], nozzle speed [11], raster pattern [15,16], and infill density [17].

Among the various available techniques in AM, FDM is one of the processes that employs thermoplastic polymers as its feedstock for 3D printing [18]. Due to their performance-to-cost ratio and ease of production, application of thermoplastic polymers in 3D printing is rapidly increasing [19]. In FDM, among the thermoplastic plastic polymers, acrylonitrile-butadiene-styrene (ABS) and polylactic acid (PLA) are the most-often used. However, on the other hand, semi-crystalline polymers are gaining traction due to their superior impact strength, chemical resistance, excellent mechanical characteristics at high-temperature environments, and wear resistance properties [18,20]. Despite their advantages, semi-crystalline polymers are challenging to use due to the volumetric shrinkage caused due to the degree of crystallinity, warpage, and anisotropic behavior compared with the amorphous polymers [18,21].

Compared to semi-crystalline polymers, a significantly high number of studies have been conducted on amorphous polymers such as ABS and PLA [18]. Furthermore, only a very few studies have concentrated on the effect of ambient temperature on the overall quality of the parts printed using FDM [13]. Although in the literature it has been reported that ambient temperature does not have any considerable effect [22], it has also been stated that increasing ambient temperature can lead to a decrease in in-built residual stresses, allowing homogenous heat transfer enabling better bonding characteristics between the filaments/roads, resulting in improved mechanical properties [10,23]. However, compared to amorphous polymers, in semi-crystalline polymers, decreasing cooling rate leads to promoting of crystallization leading to an increase in warpage [24].

In our previous works, FDM processing parameters such as print bed temperature, layer thickness, nozzle speed, raster pattern including ambient temperature on semi-crystalline polymer were investigated [1,25]. In this study, in order to gain a better understanding of the effect of ambient temperature on amorphous and semi-crystalline polymer towards the internal developed residual stresses and overall warpage, an in-depth analysis was performed. The polymers of the study were printed using FDM, simulated via COMSOL Multiphysics software, and validated experimentally. Since crystallinity is a significant factor in semi-crystalline polymers, crystallization kinetics that was developed by Levy A. was modified and incorporated into this study for semi-crystalline polymer analysis [26,27]. Along with the crystalline kinetics, the thermo-mechanical properties were also taken into consideration.

## 2. Materials and Methods

In this study, isotactic polypropylene (PP) (3D Fila, Essex, UK) was selected as a material of study along with acrylonitrile butadiene styrene (ABS P400) (UL Prospector, Overland Park, KS, USA). PP was selected in order to study the effects of ambient temperature on semi-crystalline polymers, while ABS P400 was selected for analyzing the behavior of amorphous polymer under various ambient temperatures during the FDM process. Moreover, ABS P400 and PP are commercially the most-used polymers as feedstocks in FDM; therefore, they were selected for this study [1,28,29]. These samples were printed and simulated under the following conditions: bed temperature 100 °C, line (90, 90) raster pattern, nozzle speed of 30 mm/s, and layer thickness of 0.5 mm using a nozzle diameter of 0.8 mm with an infill of 100% in order to assess the resulting warpage from the printed samples. The samples were sliced using Cura version 4.8 (Ultimaker Cura, Framingham, MA, USA), then printed using the modified Ultimaker 2, and simulated on COMSOL Multiphysics software (COMSOL, Cambridge, UK). The warpage from the printed samples was measured using an Absolute arm (8525 model) with a RS6 scanner (GH Inspection LTD, Cambridge, UK) for higher accuracy.

The extrusion temperature and ambient temperature for ABS P400 and PP are presented in Table 1 and Table 2. The term ambient temperature used in this study is also referred to as chamber temperature/environmental temperature/envelope temperature in other studies. The respective ambient temperatures considered for each sample were maintained throughout their print.

In Table 1, ABS-50 represents the ABS P400 sample that was maintained with an ambient temperature printing condition of 50 °C. Similarly, ABS-75 and ABS-120 illustrate ABS P400 samples printed at 75 °C and 120 °C ambient temperature. Correspondingly, in Table 2, PP-50, PP-75, and PP-120 depict PP samples printed at 50 °C, 75 °C, and 120 °C ambient temperature conditions.

The material properties of amorphous polymer (ABS P400) and semi-crystalline polymer are presented in Table 3 and Table 4.

In Table 4, *C_p_*, *λ*, and *ρ* for both amorphous and crystalline region are considered with respect to the simple mixing rule [1]. Here, the terms a and sc illustrate the amorphous and crystalline regions in the semi-crystalline polymer.

## 3. Modeling

**Figure 1:**
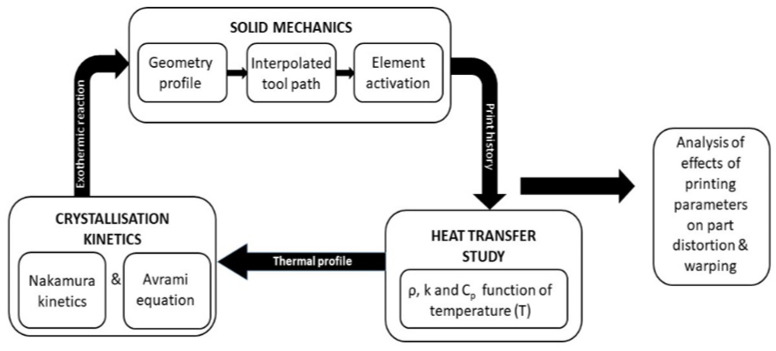
Simulation plan for predicting the part distortion in semi-crystalline polymer.

The multi-physics’ simulation study presented here incorporates various physics such as solid mechanics, heat transfer, and crystallization physics, coupled with the temperature gradient of the model, as represented in Figure 1. Due to this coupling, the incorporated physics in this simulation are temperature driven. In addition, factors such as the effect of gravity on the deposited melt, phase transition from liquid to solid, heat transfer between the deposited filaments (roads and layers), raster pattern, print bed temperature, ambient temperature, viscoelasticity, and the thermo-mechanical characteristics of the semi-crystalline polymer were considered. Due to the complexity of the incorporated physics and factors accounted, samples of dimension 50*50*2 mm were printed and simulated. The various physics incorporated in this simulation study are explained below.

### 3.1. Solid Mechanics

**Figure 2:**
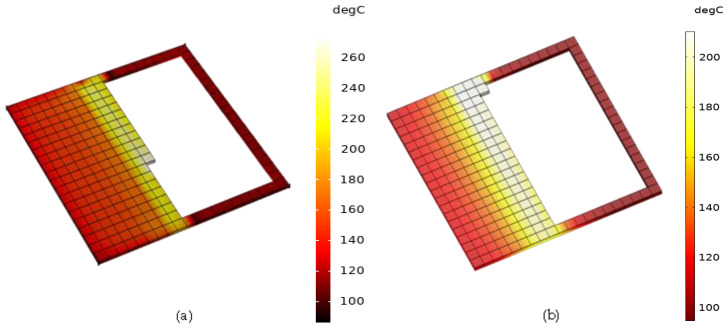
Representation of element activation from (**a**) ABS P400 and (**b**) PP samples with respect to material deposition similar to FDM process.

In order to replicate the material deposition FDM process, element activation is a commonly used method [1,3,34]. In this study, the elements in the model were activated with respect to the material deposition similarly to the FDM deposition process, as illustrated in Figure 2. Initially, the developed model was meshed with respect to the size of the filament deposited from the nozzle (0.5 mm in this study). Once the elements were meshed, they were sequentially activated with respect to the raster pattern considered in the simulation. Similar to the FDM deposition process, the elements were activated sequentially in the x and y axes; once the layer was deposited, the elements were activated in the z-axis for the next layer deposition.

Followed by the deposition process, during cooling, the FDM printed samples gradually warped with respect to the processing conditions. In order to reproduce this phenomenon, a spring foundation was used between the print bed and the simulated model, as this boundary condition allowed the model to warp freely when being cooled down [1,25,35]. Throughout the simulation, the print bed temperature was fixed at its respective temperature for each model and maintained as constant.

### 3.2. Heat Transfer Physics

Thermodynamics plays a vital role in influencing the heat transfer between the layers and the overall cooling rate of the polymer, thus also controlling the degree of crystallinity of semi-crystalline polymers. Therefore, in order to simulate the effects of printing conditions in the FDM process, it is imperative to consider factors such as thermodynamics as the material properties of polymer are temperature dependent [36,37]. This has also been corroborated by other research studies found in the literature [13,38]. Furthermore, the temperature gradient of a printed sample is also influenced by the various printing conditions, which can affect the resulting part distortions [13,39]. As aforementioned, in this study the thermo-mechanical properties of PP were expressed as a function of temperature (*T*), and the physics were coupled with respect to the thermal gradient of the model [36,37]. The general energy balance used for heat transfer is given in Equation (1):

(1) $$\rho C_{p}\frac{\partial T}{\partial t}-\nabla \cdot \left(\lambda \nabla T\right)=Q$$

In Equation (1), ρ is the density, $C_{p}$ is the specific heat capacity, and λ represents the thermal conductivity of the polymer and *Q* denotes the heat source.

### 3.3. Crystallization Kinetics’ Physics

It has been well established in the literature that crystallization kinetics is highly temperature dependent and, thus, with the change in thermal gradient during the printing, crystallization can severely affect the properties of the printed semi-crystalline polymer [40]. Therefore, along with the thermal history of the printed model, the semi-crystalline polymer model (PP) considered in this study was also driven by the polymer crystallization kinetics. This was achieved by modifying and incorporating the crystallization physics that was developed by Levy [26,27]. The thermo-mechanical properties of PP were expressed as a function of temperature (T), as was reported in the past that thermo-mechanical properties (ρ, $C_{p}$, λ) are highly influenced by the temperature gradient of the system and the degree of crystallization [25,36].

In order to calculate the change in crystallinity with respect to time under non-isothermal crystallization conditions, Nakamura extended the Avrami equation, which is expressed as [1,25]:

(2) $$\alpha \left(t\right)=1-exp{\left[-\overset{t}{\underset{0}{\int }}K\left(T\right)dt\right]}^{n}$$

Here, *t* is time, n is the Avrami index, and *K*(*T*) represents the Nakamura crystallization kinetics’ function derived from Avrami’s isothermal kinetics. Koscher et al. performed DSC experiments for iso-thermal and non-iso thermal conditions and proposed *K*(*T*) as [26,36,37]:

(3) $$K\left(T\right)={\left(\frac{4}{3}\pi N_{0}\left(T\right)\right)}^{\frac{1}{3}}G_{0}\times \exp\left(-\frac{U^{*}}{R\left(T-T_{\infty }\right)}\right)exp\left(-\frac{K_{g}}{T\left(T_{f}-T\right)}\right)$$

The Nakamura crystallization kinetics’ model, which was incorporated in this study, is a widely used equation for simulating crystallization in a semi-crystalline polymer as it considers both time (*t*) and temperature (*T*) as driving factors [26,41,42]. Please refer to our previous work for the detailed explanation of all the incorporated boundary conditions, equations used for expressing material properties of PP as a function of temperature, and physics’ couplings [1].

It should be noted that for ABS P400 and PP similar boundary conditions were used in solid mechanics and heat transfer physics. Due to the crystallization kinetics, crystallization physics was incorporated in PP models and coupled with the other physics through the function of temperature (*T*).

## 4. Results and Discussion

**Figure 3:**
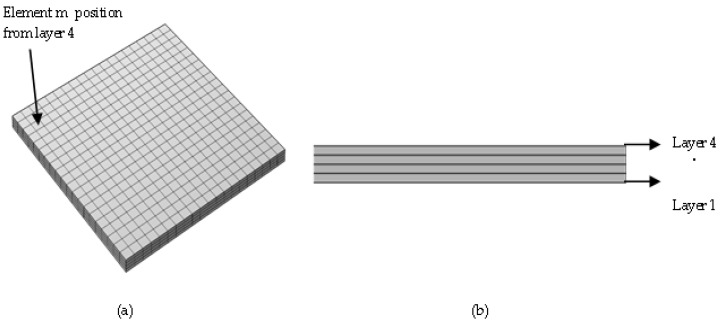
(**a**) Location of element m and iso-metric view of the sample (ABS P400 and PP). (**b**) Side view of the sample illustrating the layer sequence.

In order to analyze the effect of various ambient temperature conditions considered, an element was selected from the top layer of the printed/simulated samples. This element was selected from the co-ordinates 7.8, 2.1, and 1.5 mm and will be referred to as element m throughout the study. Element m was specifically selected from the top layer of the printed/simulated samples due to their prolonged exposure to the ambient temperature. Figure 3 represents the position of element m in an isometric view along with the side view of the modeled samples.

### 4.1. Temperature Distribution

**Figure 4:**
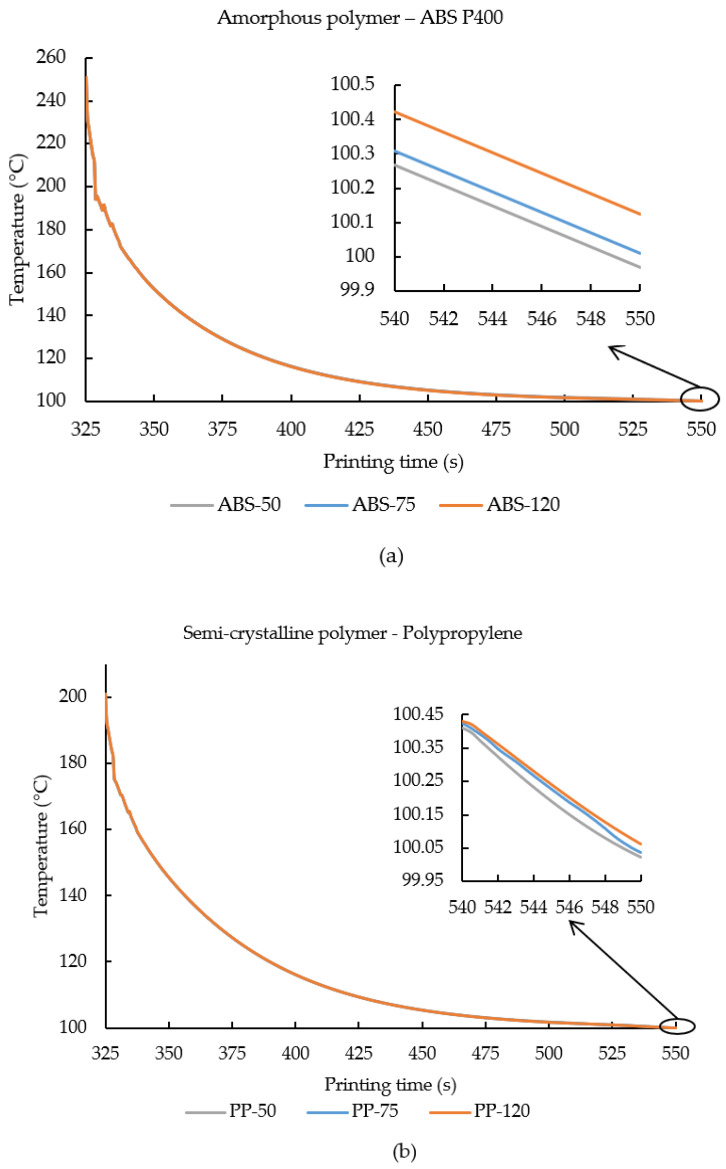
Thermal history of element m is plotted against the printing time for (**a**) ABS P400 and (**b**) PP samples printed under various ambient temperature printing conditions. An inset plot is provided in order to signify the effect of ambient temperature on the temperature gradient of element m.

In this study, ABS P400 was extruded at 270 °C and deposited on the bed temperature of 100 °C, while polypropylene (PP) was extruded at 210 °C and deposited on the bed temperature of 100 °C. Once the polymers were extruded from the nozzle, they were allowed to cool down to reach the bed temperature. Here, Figure 4a,b shows the cooling curve of element m from both ABS P400 and PP under various ambient temperature conditions considered in this study. In Figure 4a,b, since element m was selected from the top layer (layer 4), the x-axis (printing time) begins at 325 s. Due to the substantial temperature difference between the extrusion temperature and the print bed temperature, the impact of change in ambient temperature is not apparent from the graphs (Figure 4a,b). Therefore, an inset plot is presented where effect on ambient temperature on the cooling curves of the polymers can be seen clearly.

From Figure 4a, it can be seen that element m deposition temperature starts at 251 °C (below 270 °C). This is mainly because, when element m was deposited on the third layer, the temperature of the third layer and the loss of energy through the surrounding due to the maintained ambient temperature decreases the recorded deposition temperature to 251 °C. Similarly, this phenomenon can also be observed in Figure 4a, where, even though PP was extruded at 210 °C, the recorded temperature is around 201 °C. After the deposition, both the polymers were allowed to cool to reach the bed temperature (100 °C).

The inset plots from Figure 4a,b, show that with an increase in ambient temperature the cooling rate decreased. Since element m was selected from the top layer (layer 4), after deposition the printing process was terminated and the sample was allowed to cool. At 540–550 s, an inset plot is provided in Figure 4a,b to pronounce the effects of ambient temperature on the cooling rate of the printed polymers (amorphous and semi-crystalline) in FDM. From the graphs (Figure 4a,b), one can see the cooling rate of ABS-120 and PP-120 was slower than ABS-75 and PP-75, while the cooling rate of ABS-75 and PP-75 was slightly slower than ABS-50 and PP-50. It is evident from Figure 4a,b that with increase in ambient temperature the cooling rate of the amorphous and semi-crystalline polymers decreased considerably.

### 4.2. Evolution of Residual Stresses

**Figure 5:**
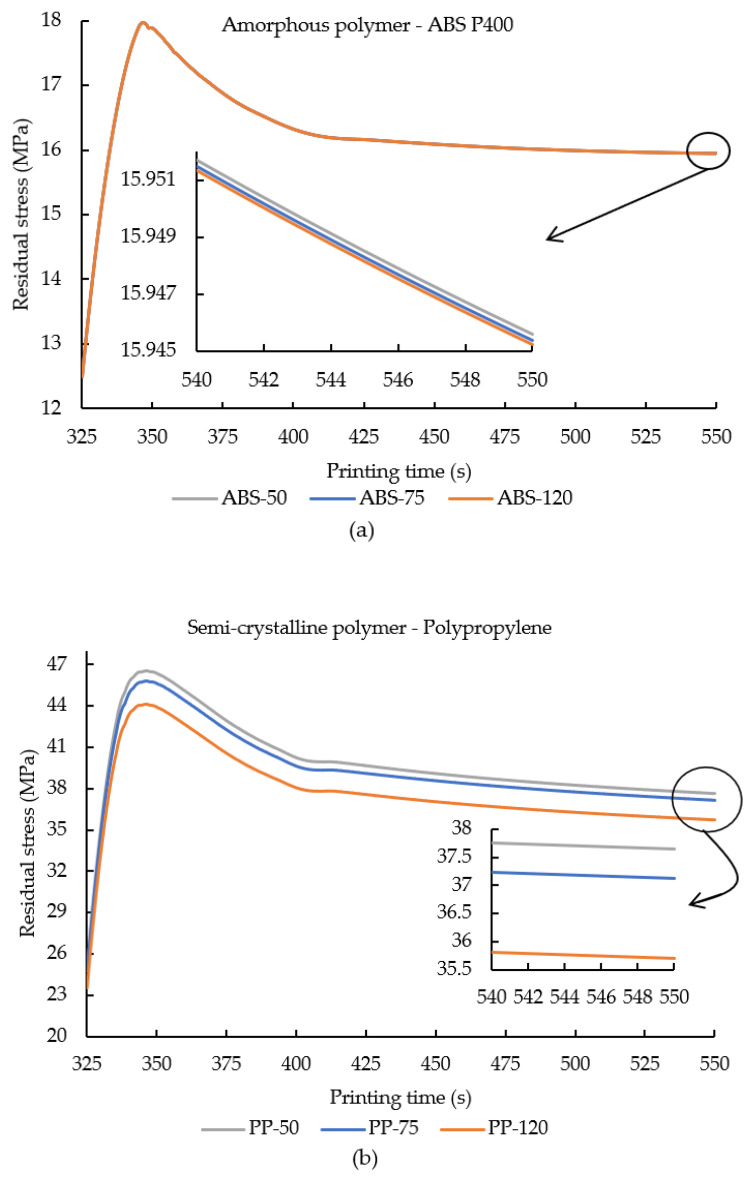
Residual stress values from element m plotted against the printing time for (**a**) ABS P400 and (**b**) PP samples printed under various ambient temperature printing conditions. An inset plot is provided in order to signify the effect of ambient temperature on the internally developed residual stresses at element m.

Residual stresses in polymers were affected by the in-built thermal residual stresses during the printing process due to non-uniform temperature distribution resulting in anisotropic cooling [38,43]. Figure 5a,b illustrates the evolution of residual stress from element m plotted against the respective printing time of the samples. An inset plot is presented (at a similar printing time) to signify the effects of increase in ambient temperature towards residual stresses in the printed polymers.

Figure 5a,b depicts that, on initial stages of deposition of element m, the residual stress in amorphous and semi-crystalline polymer increased significantly before reaching the equilibrium state. Residual stress was induced in FDM printed polymers due to the trapped thermal residual stresses during the continuous printing process due to the non-homogenous cooling [44]. Additionally, in semi-crystalline polymers, due to volumetric changes (influenced by the crystallization phenomenon), the developed residual stress was considerably higher than the stress accumulated in amorphous polymers (ABS P400 in this case) [25].

In order to achieve a better understanding between the development of residual stress in amorphous and semi-crystalline polymers and to emphasize the influence of crystallization on residual stress and resulting warpage in semi-crystalline polymers, here a comparison between ABS P400 and PP samples was made. When compared with ABS-50, PP-50 showed a significant increase of 136.1% in residual stress, while a comparison between samples ABS-75 and PP-75 illustrated an increase of 132.8% in residual stress. A comparison of samples ABS-120 and PP-120 depicted an increase of 123.9% in residual stress. These comparisons revealed that the semi-crystalline polymers in general (PP in this case) displayed higher residual stress accumulation when compared with amorphous polymers. Moreover, it was also evident that, with an increase in ambient temperature in both ABS P400 (amorphous polymer) and PP (semi-crystalline polymer), the developed residual stress decreased continuously.

In Figure 5a it can be seen that increasing ambient temperature decreased the accumulated residual stresses. Increasing the ambient temperature from 50 °C to 75 °C demonstrated a small decrease of 0.22 KPa in residual stress. On a further increase in ambient temperature, from 75 °C to 120 °C, a further decrease of 0.37 KPa in residual stress was noticed. Even though an increase in ambient temperature led to a drop in residual stress, no significant difference was seen here. However, a considerable drop in residual stress in semi-crystalline polymer (PP) was observed upon increasing the ambient temperature (Figure 5b). Similar to ABS P400, when the ambient temperature was increased, a decrease in the accumulation of residual stress was noticed (Figure 5b). Here, increasing ambient temperature from 50 °C to 75 °C showed a decrease of 1.4% accumulated residual stress. On a further increase in ambient temperature to 120 °C, the drop in residual stress observed increased further to 5.2%. In studies conducted by Ferreira et al. [45] and Zhang et al. [46], researchers reported that an increase in ambient temperature enhanced the uniform temperature distribution between the filaments and layers, thereby improving the bonding characteristics and decreasing the likelihood for accumulation of thermal residual stresses in the printed samples.

### 4.3. Resulting Warpage

**Figure 6:**
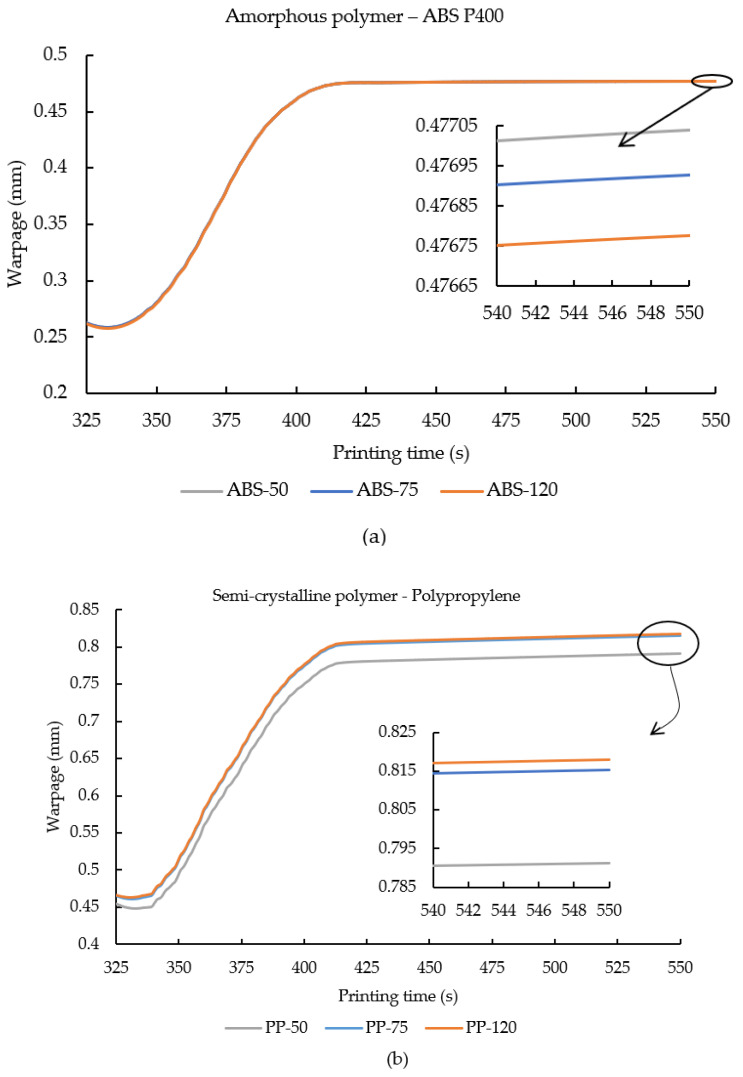
Warpage results from element m plotted against the printing time for (**a**) ABS P400 and (**b**) PP samples printed under various ambient temperature printing conditions. An inset plot is provided in order to signify the effect of ambient temperature on the resulting warpage from element m.

Warpage is a product of the accumulated thermal residual stresses in the FDM printed samples and degree of crystallization specifically in semi-crystalline polymers [47,48]. In Figure 6a,b, warpage results from the simulated samples are plotted against their printing time in order to compare and contrast the effect of an increase in ambient temperature over warpage of the 3D printed samples. Similar to the other results presented in this study, an inset plot is attached to Figure 6a,b to magnify the effects of ambient temperature on warpage.

Figure 6a,b depicts a linear increase in warpage along with the ABS P400 and PP samples’ respective printing/cooling time. During the initial deposition phase of element m, it can be seen that the warpage trend was flat followed by a gradual increase and the samples were cooled. This flat region was observed due to the settling of element m after being deposited from the nozzle.

From the inset plot in Figure 6a, it is observed that, with an increase in ambient temperature along with the accumulated residual stress, the resulting warpage was also reduced. On increasing the ambient temperature from 50 °C to 75 °C, an insignificant decrease of 0.02% was noticed. On further increasing the ambient temperature to 120 °C, a further insignificant decrease of 0.05% was observed. Even though the decrease in warpage seen here was less significant, it still corroborated that, with increasing the ambient temperature, residual stress and warpage decreased. On the other hand, in PP samples, when the ambient temperature was increased, on the contrary to decreases in residual stress, here an increase in warpage was observed. When the ambient temperature was increased from 50 °C to 75 °C, an increase of 3% warpage was noticed. On further increasing the ambient temperature to 120 °C, a warpage rise of 3.4% was seen. Here, an increase in warpage in PP samples can be explained by taking into consideration the crystallization kinetics in semi-crystalline polymers. As aforementioned in Section 4.1 (temperature distribution), increase in ambient temperature decreased the cooling rate of the printed samples, thus promoting uniform temperature distribution throughout the printed sample, enhancing the bonding between the filaments and the layers [25]. A slow cooling rate and homogenous temperature gradients favor crystallization in semi-crystalline polymers. Increasing the ambient temperature printing condition led to an increase in crystallization in the polymer molecules of the printed samples. During crystallization, semi-crystalline experienced volumetric contraction due to the realignment of the polymer molecules; a higher degree of crystallization led to greater volumetric contraction [49,50]. Thus, increasing the ambient temperature promotes homogenous cooling and enhances the degree of crystallization of PP-50, 75, and 120, thereby resulting in an increase in warpage.

### 4.4. Relation between Final Residual Stress and Overall Warpage

**Figure 7:**
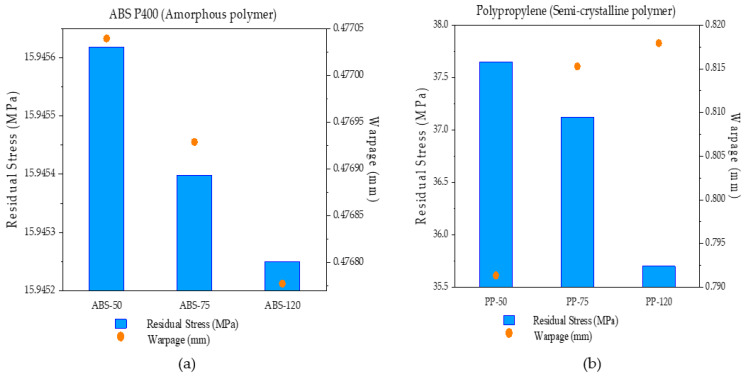
Comparison of final residual stress and overall warpage from element m of (**a**) ABS P400 and (**b**) PP samples simulated at various ambient temperature printing conditions.

In order to gain further insight towards the relationship between residual stress and warpage, both ABS P400 and PP samples were plotted against their final residual stress and overall warpage results in Figure 7a,b. Even though these results were already presented in previous sections (on warpage and residual stress), here, the overall values of residual stress and warpage are plotted against each other to pronounce the relationship between them and to emphasize the importance of crystallinity in semi-crystalline polymers.

From Figure 7a,b, it is evident that with an increase in the ambient temperature printing condition, the residual stress was continuously decreasing in both amorphous and semi-crystalline polymer models. Samples ABS-50 and PP-50 showed high residual stress, while samples ABS-75 and PP-75 exhibited moderate residual stress out of the samples. Among all the samples, ABS-120 and PP-120 displayed the least amount of in-built residual stress. However, in terms of warpage, only ABS samples showed a decrease in warpage along with the drop in residual stress on increasing the ambient temperature. Sample ABS-50, similar to exhibiting high residual stress among the studied samples, showed high warpage, while ABS-75 indicated moderate warpage and ABS-120 demonstrated minimum warpage along with the predicted residual stress results. On the other hand, increasing the ambient temperature seemed to have increased the resulting warpage in PP samples. Among all the samples, sample PP-50 with maximum residual stress also presented minimum warpage. On increasing the ambient temperature, sample PP-75 showed an increase in warpage while the in-built residual stress was decreased. Lastly, sample PP-120 showed the highest warpage while presenting the lowest residual stress in polypropylene samples. It can be clearly seen here that, unlike the amorphous polymer samples, semi-crystalline polymer samples, despite a decrease in residual stress, still showed a significant increase in warpage. This increase in warpage can be related to the volumetric contraction contributed by the crystallization in semi-crystalline polymers, where an increase in ambient temperature promoted and increased the degree of crystallization in semi-crystalline polymers, thereby resulting in high warpage.

### 4.5. Experimental Validation

In order to validate the warpage results from the simulated samples, ABS P400 and PP samples were printed under similar printing conditions using modified Ultimaker 2 and 3D scanned using an Absolute arm (8525 model) with a RS6 scanner for higher accuracy. In order to measure the warpage from element m of the printed samples, the samples were scanned into 3D CAD models and a cartesian co-ordinate was formulated at (0, 0, 0) while another axis was constructed at the location of element m (7.8, 2.1, 1.5). The deviation observed between the nominal axis (0, 0, 0) and the axis formulated at element m location (7.8, 2.1, 1.5) was measured as the warpage value of the respective samples.

From Table 5, it can be seen that the warpage results reported from the developed model are in very good agreement with the experimentally measured warpage values. In Table 5, due to the miniscule difference found in warpage values, only ABS-50 was validated and reported here. Similarly, since only a small difference was observed from the warpage values between PP-75 and PP-120, only PP-120 warpage values are presented in Table 5.

**Figure 8:**
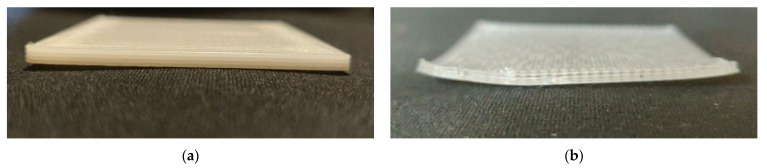
Comparison of warpage between FDM printed ABS P400 (**a**) and PP (**b**) samples.

Figure 8 is presented to illustrate the difference in warpage between the ABS P400 and PP printed sample. Since the warpage difference between ABS P400 and PP samples printed at various ambient temperatures only varied slightly, samples printed at 120 °C are shown in Figure 8. From Figure 8, it can be seen that sample PP-120 warped significantly more in comparison to sample ABS-120.

## 5. Conclusions

In this study, an in-depth analysis on the effect of ambient temperature on the internally developed residual stresses and resulting warpage in acrylonitrile-butadiene-styrene (ABS) and polypropylene (PP) was performed. The developed 3D model was incorporated with a custom, modified crystalline physics for considering the crystalline nature for the semi-crystalline polymer under study. The thermo-mechanical material properties of polypropylene were also expressed in terms of function of temperature as they are highly temperature dependent. The various physics invoked in this simulation study were coupled with respect to temperature, allowing the model to change with respect to the temperature gradient.

From the study, when the ambient temperature was increased from 50 °C to 75 °C and further to 120 °C, there was an insignificant reduction (<1%) in residual stress and warpage in ABS, while in PP, there was a considerable decrease of 1.4% and 5.2% observed in residual stresses. In terms of warpage, conversely, increases of 3% and 3.4% were noted. Therefore, it can be concluded that increasing ambient temperature results in a decrease in residual stress in both ABS and PP. An increase in ambient temperature leads to homogenous cooling of the printed/simulated part, also improving bonding between the deposited polymer filaments and roads. The results show that for ABS, with an increase in ambient temperature, both residual stress and warpage decrease steadily. However, for polypropylene, even with increase in ambient temperature, the residual stress decreased; on the other hand, there was a continuous increase in warpage. This increase in warpage can be explained due to the slow cooling rate of the semi-crystalline polymer, which leads to an increase in crystallization, thus resulting in a high amount of warpage.

## 6. Future Work

In the future, the simulation of 3D printing of various types of amorphous and semi-crystalline polymers will be investigated.

## Figures and Tables

**Table 1:** Processing parameters for ABS P400.

Processing Conditions	Extrusion Temperature (°C)	Ambient Temperature (°C)
ABS-50	270	50
ABS-75	270	75
ABS-120	270	120

**Table 2:** Processing parameters for PP.

Processing Conditions	Extrusion Temperature (°C)	Ambient Temperature (°C)
PP-50	210	50
PP-75	210	75
PP-120	210	120

**Table 3:** Material properties of ABS P400 [28,29,30,31,32].

Material Parameters	Values
Thermal conductivity, *k* (W/m K)	0.1777
Specific heat capacity, $C_{p}$ (J/kg K)	2080
Density, *ρ* $(\mathrm{kg}/m^{3}$)	1050
Co-efficient thermal expansion, CTE *α* (K^−1^)	10.08 × 10^−5^
Young’s modulus, E (GPa)	2.65
Glass transition temperature, $T_{g}$ (°C)	94

**Table 4:** Material properties of PP [33]. Reprinted with permission from [33]. Copyright 2005 Le Goff R.

Thermal Property for Amorphous (a) and Semi-Crystalline (sc) States	Numerical Equation
*C*_pa_ (*α*, *T*)	3.1 *T* + 2124
*λ*_a_ (*α*, *T*)	−6.25 × 10^−5^ *T* + 0.189
*ρ*_a_ (*α*, *T*)	1/(1.138 + 6.773 × 10^−4^ *T*)
*C*_psc_ (*α*, *T*)	10.68 *T* + 1451
*λ*_sc_ (*α*, *T*)	−4.96 × 10^−4^ *T* + 0.31
*ρ*_a_ (*α*, *T*)	1/(1.077 + 4.225 × 10^−4^ *T*)

**Table 5:** Comparison of warpage values of simulated and experimental ABS and PP samples from element m.

Samples	Predicted Warpage (FEA) (mm)	Measured Warpage (Experimental) (mm)
ABS-50	0.477	0.501
PP-50	0.791	0.82
PP-120	0.818	0.84

## Data Availability

The data presented in this study are available on request from the corresponding author.
